# Study on the Electro-Optical Properties of Polymer-Dispersed Liquid Crystals Doped with Cellulose Nanocrystals

**DOI:** 10.3390/molecules30153273

**Published:** 2025-08-05

**Authors:** Jiayan Wang, Yan Qiao, Ziyi Yang, Yue Han, Hui Zhang, Zhiguang Li, Guili Zheng, Yanjun Zhang, Lizhi Zhu

**Affiliations:** 1 Arizona College of Technology, Hebei University of Technology, Tianjin 300401, China; wjy20030316@163.com (J.W.); 13230068133@163.com (Y.Q.); ziyiyang1004@163.com (Z.Y.); 2 School of Science, Hebei University of Technology, Tianjin 300401, China; hycreative@163.com (Y.H.); zhanghui@hebut.edu.cn (H.Z.); zhglee@hebut.edu.cn (Z.L.); zhengguili@hebut.edu.cn (G.Z.); 3 College of Light Industry Science and Engineering, Tianjin University of Science and Technology, Tianjin 300457, China

**Keywords:** polymer-dispersed liquid crystals, cellulose nanocrystals, electro-optical properties

## Abstract

The present study focuses on the effect of doping KH560-modified cellulose nanocrystals (CNCs) on the electro-optical characteristics of polymer-dispersed liquid crystals (PDLCs). PDLC films were fabricated through the polymerization-initiated phase separation (PIPS) process and doped with CNC nanoparticles at various concentrations. At low concentrations, the CNCs at the interface, by virtue of their unique chiral characteristics, induce an orderly arrangement of liquid crystal molecules. Meanwhile, the interaction between the film’s fiber structure and the liquid crystal droplets brings about an augmentation in the arrangement efficiency. The excellent dispersion of CNCs diminishes the random alignment of liquid crystal molecules and mitigates light scattering. Additionally, it aids in the deflection of the liquid crystal director, facilitating the lubrication of the liquid crystals’ movement. It is remarkable that within the range of relatively lower CNCs doping concentrations, specifically from 0.005 wt% to 0.05 wt%, the PDLC films exhibit lower threshold and saturation voltages, faster response, enhanced viewing angle performance and higher contrast.

## 1. Introduction

Polymer-dispersed liquid crystals (PDLCs) are composites with unique electro-optical properties, consisting of liquid crystal droplets dispersed in a polymer matrix. The material exhibits a remarkable capability of rapidly transitioning between transparent and opaque states by means of an applied electric field, and its core principle of operation is based on the change in the arrangement of the liquid crystal molecules under the action of an electric field. In the absence of an electric field, the liquid crystal droplets in PDLCs are randomly oriented, leading to light scattering and an opaque state, whereas when an electric field is applied, the liquid crystal molecules are arranged in an orderly manner along the direction of the electric field, which reduces light scattering and transforms the material into a transparent state [1]. The potential for high brightness modulation without the use of a polarizer signifies the wide range of applications of PDLCs, particularly in smart dimming windows [2,3,4], variable light attenuators [5,6,7], sensors [8,9,10], holographic gratings [11,12,13] and displays [14,15,16].

Research on the application of doping technology in PDLCs has demonstrated that the electro-optical properties of PDLCs can be substantially enhanced by the incorporation of nanoparticles, dyes, chiral agents and other materials. Notably, it has been shown that doping with silica (SiO_2_) nanoparticles is capable of decreasing the driving voltage and improving the arrangement of liquid crystal molecules in PDLC microdroplets [17]. Moreover, doping indium tin oxide (ITO) nanoparticles has been found to enable PDLC to have a lower driving voltage and higher contrast [18]. Furthermore, the response time of PDLC films doped with MgO nanoparticles has been shown to be accelerated [19]. The dopant metal nanoparticles (MNPs) can also modify the electro-optical properties of PDLCs. For example, Ahmad et al. demonstrated that doping gold nanoparticles (GNPs) enables PDLCs to achieve a lower driving voltage and higher contrast ratio (CR) [20]. Shriyan et al. demonstrated that doping multiwalled carbon nanotubes (MWCNTs) in PDLCs improves the frequency response speed [21]. In addition, doping organic dyes, such as anthraquinone dyes, has been shown to exhibit a high contrast ratio [22]. Katariya-Jain et al. doped azo dyes into PDLC films, which significantly improved contrast, lowered the threshold and saturation voltage and provided additional advantages in color control due to their high absorption properties and alignment ability under an electric field [23]. Specific compounds can likewise be used as dopants to improve the electro-optical properties of PDLCs. Terpene alcohols represent a class of hydrocarbons that are derived from isoprene. Huang and his team found that doping a moderate amount of geraniol (GOL) in PDLC can significantly reduce the driving voltage and maintain a high contrast ratio [24]. The addition of chiral dopants and polymer thiol materials to PDLC has also been demonstrated to affect its electro-optical properties. Sun et al. reduced the initial light scattering transmittance of PDLC films by adding chiral dopant S811 and achieved lower driving voltage and higher contrast in the PDLC system by adding polymer thiol material Capcure 3-800 [25].

In recent years, cellulose nanocrystals (CNCs) have emerged as a prominent research focus, owing to their distinctive physical, chemical and biological characteristics. As a natural polymer extracted from renewable resources, CNCs have become an ideal dopant material for enhancing the structural stability and improving the electrical conductivity of composites by virtue of their nano-size, high Young’s modulus, high specific surface area and high mechanical strength [26,27,28]. CNCs can be combined with various matrix materials through surface modification, enriching their surface chemical structure and endowing composite materials with unique functional properties [29]. For instance, CNT/polyaniline (PANI) composites doped with CNCs can improve the stability and conductivity of the material, thus successfully developing methanol gas sensors with high selectivity and sensitivity [30]. Additionally, the application of CNCs in photocatalytic materials has also shown potential, especially when combined with zirconium (Zr) and MnO_2_ nanorods, which significantly improve the photocatalytic efficiency and antibacterial performance of dye degradation [31]. CNCs have also been utilized to significantly enhance microwave absorption properties through surface modification with cobalt particles, which is particularly suitable for electromagnetic interference suppression in 5G technology [32]. Within the textile domain, it has been established that the doping of CNCs with sodium carbonate is conducive to enhancing the mechanical and refractory attributes of alginate fibers. Consequently, these fibers emerge as a preferable option for the production of refractory textile materials [33]. The application of nanofibrous materials as a dopant in polymer–liquid crystal composite systems has been reported in previous studies. Miao et al. incorporated gelatin nanofibers as carriers loaded with CeO_2_ nanoparticles into the PDLC matrix via electrospinning technology. The gelatin nanofiber network effectively addressed nanoparticle agglomeration issues due to its high specific surface area and porosity. Meanwhile, the CeO_2_ nanoparticles regulated the polymerization process through their UV absorption properties, reducing the driving voltage while enhancing contrast [34]. Deng et al. significantly improved the electro-optical performance of PDLC by introducing a polyvinyl alcohol (PVA) nanofiber network. The PVA nanofibers not only served as a supporting structure to modulate the polymer network morphology but also enhanced light scattering at the liquid crystal droplet interfaces, improving contrast ratio [35]. Satapathy et al. developed a polymer-stabilized liquid crystal (PSLC) system based on a biopolymer network of cellulose nanocrystals (CNCs). This system features a unique “Swiss cheese” architecture that maintains stable threshold voltage while significantly reducing the off-state response time and achieving temperature insensitivity. At a CNC content of 9.3 wt%, the device demonstrates a haze value of 67% and exhibits excellent switching contrast. This novel network structure provides a simple yet effective solution for high-performance smart windows [36].

In this paper, a PDLC doped with a KH560-modified CNC composite was successfully prepared. This work systematically investigates the influence of CNC’s chiral order-induced effects on PDLC electro-optical properties and proposes an interface lubrication mechanism to analyze response time. The experimental results show that the performance of KH560-modified CNC-doped PDLC samples is significantly improved compared to undoped samples, with a threshold voltage of 8.8±0.7 V, a saturation voltage of 14.4±0.5 V and a response time of 8±0.7 ms. Additionally, we analyzed the effects of CNC doping on the viewing angle characteristics and contrast of PDLC.

## 2. Results and Discussion

**Figure 1:**
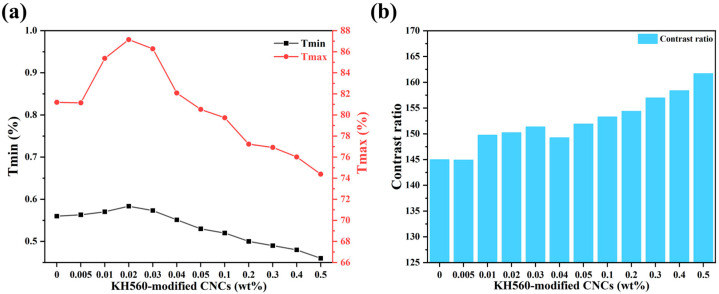
Variation of (**a**) minimum and maximum transmittance of samples and (**b**) contrast of samples.

When light is incident, Figure 1a shows the on-state transmittance and the off-state transmittance of each sample. It can be seen that the variation curves of both on-state and off-state transmittance show an upward and then a downward trend. Figure 1b shows the contrast variation of all the samples. It is clear from the figure that the overall contrast ratio shows a gradual increase. This phenomenon can be attributed to the following factors. Without CNC doping, the liquid crystal droplets in the PDLC are randomly distributed. When a small amount of CNC is introduced, its fibrous structure reduces the randomness of liquid crystal droplet distribution, effectively reducing light scattering and increasing the off-state transmittance. At the same time, due to the relatively low doping concentration, the refractive index of the polymer matrix is closely matched to that of the liquid crystal domains, thereby enhancing the off-state transmittance. Although the off-state transmittance also increases to some extent, the open-state transmittance increases more rapidly than the off-state transmittance, thereby improving the contrast ratio compared to the undoped sample. However, when the CNC concentration exceeds a critical value (0.02 wt%), the refractive index of the polymer matrix changes, resulting in a mismatch between the refractive indices of the polymer matrix and the liquid crystal domains. This triggers a significant change in the light scattering characteristics, which subsequently causes a decrease in the open state transmittance [37]. In addition, irregular clustering of CNCs may be induced in this case, and this microstructural inhomogeneity further increases the scattering loss, leading to a further decrease in the off-state transmittance. However, since the off-state transmittance decreases more rapidly, the contrast ratio still maintains the trend of continuing to increase [38].

When PDLC is in the on-state, the liquid crystal director is parallel to the direction of the electric field, and the liquid crystal microdroplets are anisotropic uniaxial crystals. When light is incident at any angle, the refractive index of the liquid crystal to the propagating light is between the ordinary light refractive index and the extraordinary light refractive index. At this time, the expression of the equivalent refractive index *n*_eff_ of the liquid crystal microdroplet is as follows [39]:

(1) $$\;\;\;\frac{1}{n_{eff}^{2}}=\frac{{cos}^{2}\theta }{n_{o}}+\frac{{sin}^{2}\theta }{n_{e}}$$

where *n*_o_ is the ordinary refractive index of the liquid crystal microdroplets, *n*_e_ is the extraordinary refractive index of the liquid crystal and *θ* is the angle between the direction of light transmission on the droplet and the direction of the droplet director (*n*_e_ direction). When visible light is incident vertically, the incident light propagates along the directors of the liquid crystal droplets; at this point, *θ* is close to 0 degrees. Therefore, according to Formula (1), the effective refractive index of the liquid crystal to the propagated light is *n*_o_, and PDLC films exhibit a transparent state. With the increase of *θ*, the effective refractive index of the liquid crystals gradually transitions from *n*_o_ to *n*_e_.

**Figure 2:**
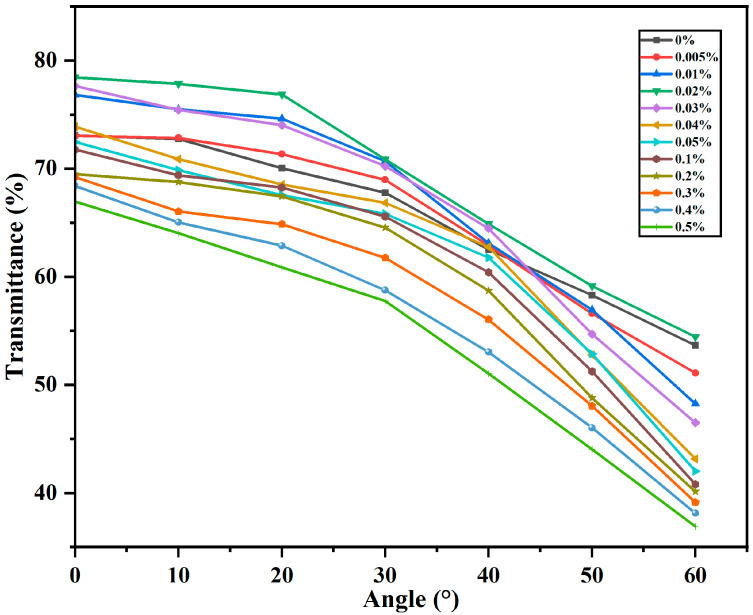
The transmittance corresponding to the saturation voltage with angle of view for samples.

Figure 2 shows the transmittance of PDLC samples with different doping concentrations in the open state at various angles. It should be noted that the refractive index of the doped CNC also plays a role in affecting the optical properties of the system. Specifically, we define the refractive index of the polymer system as *n*_p_; it refers to the cured PDLC matrix, which is calculated by mixing prepolymers in different mass percentages and determining the refractive index of the mixture based on these mixing percentages (*n*_p_ ≈ 1.516) [40]. The degree of match between the refractive index of the liquid crystal droplets and the refractive index of the polymer matrix in PDLC directly affects the transmittance [41]. (Transparent when closely matched, scattering when poorly matched.) It can be seen from Figure 2 that the transmittance increases at low doping concentrations, which indicates that at low doping concentrations, the effective optical refractive index *n*_eff_ of liquid crystals is closer to the refractive index *n*_p_ of the cured polymer system. Furthermore, at low doping concentrations, the 0.02 wt% sample has the optimum viewing angle characteristics. At small angles of incidence, the liquid crystals are optically well matched to the polymer, so the transmittance is high. However, as the angle of incidence increases, the effective refractive index of the liquid crystals gradually transitions from *n*_o_ to *n*_e_, and the difference between the refractive index of the liquid crystals and the refractive index n_p_ of the polymer system gradually increases, which significantly reduces the optical match and leads to a rapid decrease in transmittance. Under the condition of higher doping concentration, the matching between *n*_p_ and *n*_o_ decreases compared to low-concentration doping, and therefore the transmittance is lower at the initial small angle. As the angle of incidence increases, the effective refractive index of the liquid crystals becomes less matched to the polymer [42].

**Figure 3:**
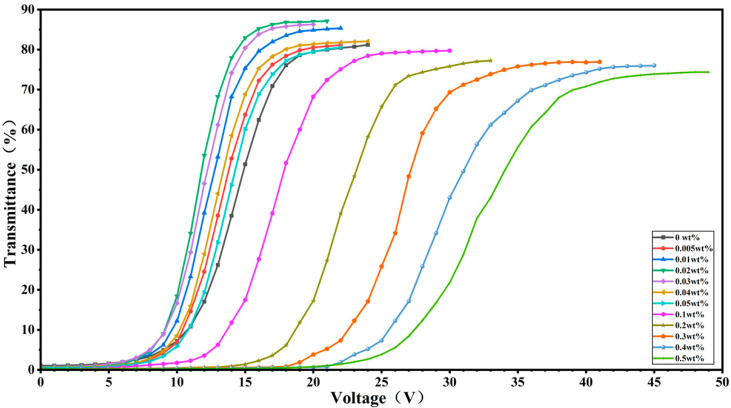
The voltage–transmittance curve of samples.

Figure 3 shows the electro-optical curves of all PDLC samples. For low CNC doping concentrations (0.005–0.05 wt%), the electro-optical curves of the PDLCs are shifted to the left compared to the undoped E7 PDLC. Conversely, for higher doping concentrations (0.1–0.5 wt%), these curves shift right relative to the undoped ones. This indicates that doping a small amount of CNC significantly reduces the threshold and saturation voltages of the PDLC devices.

**Figure 4:**
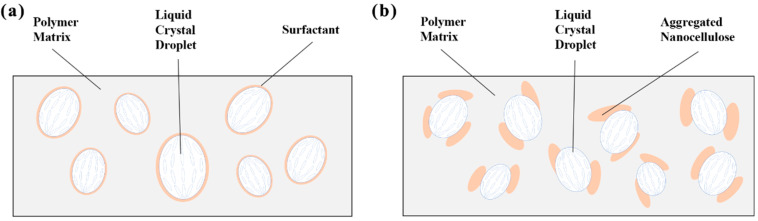
(**a**) The mechanism diagram of CNC acting as a lubricant. (**b**) CNC aggregation at the boundaries.

When a small amount of KH560-modified CNCs was doped into the PDLC system, the modified CNCs formed stable interactions with the polymer matrix through their silicon–oxygen bonds (Si-O-C). This interaction promotes the homogeneous dispersion of KH560-modified CNCs in the polymer. At the same time, the silanol group (-Si(OH)_2_) is able to balance the lipophilic properties to form a unique hydrophilic–lipophilic character. This amphiphilic property enables KH560-modified CNCs to disperse at the boundary between liquid crystals and polymers spontaneously. Significantly improves dispersion in lipophilic polymer matrices, as shown in Figure 4a. This mechanism is like the effect of doping other surfactants [43,44].

**Figure 5:**
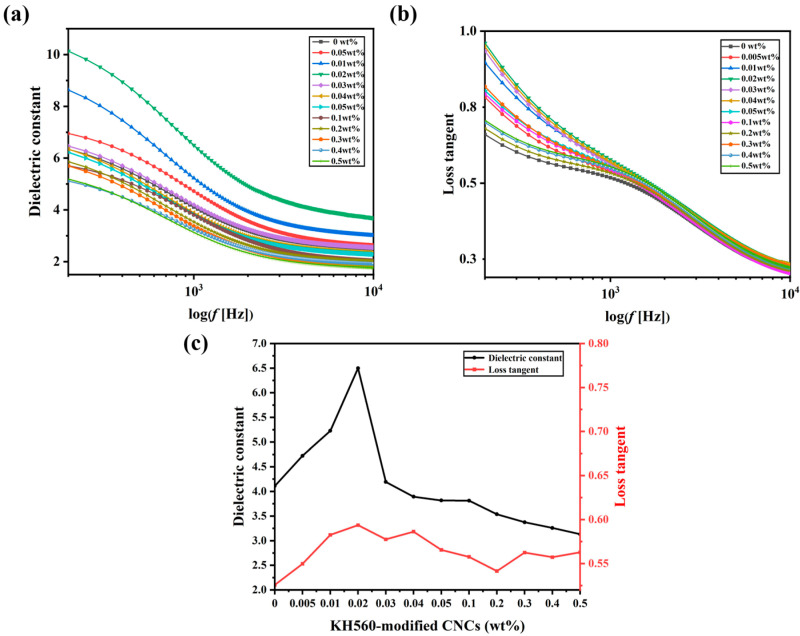
Frequency dependence of (**a**) dielectric constant, (**b**) loss tangent, (**c**) dielectric constant and dielectric loss tangent at 1 kHz of PDLCs with different KH560-modified CNCs concentrations.

Figure 5 shows the variation of the dielectric constant and loss tangent with frequency for all samples at 1v. This allows characterizing the effect of CNC doping on the intrinsic state of the sample when liquid crystal microdroplets are randomly oriented.

Doping at a low concentration can significantly enhance the overall dielectric constant of PDLCs. Because the uniformly dispersed KH560-modified CNCs increases the effective contact area with the substrate, and under the action of the electric field, orientational polarization will occur to promote interfacial polarization [45]. At the same time, the KH560-modified CNCs at the interface may also inhibit the charge leakage by reducing the interfacial defects, which ultimately synergistically enhances the dielectric constant of PDLCs [46,47]. However, it will accumulate charges at the interface, leading to the enhancement of the dielectric loss.

With the increase in doping concentration, the CNC with a hydroxyl group will agglomerate (Figure 4b). The agglomeration leads to higher localized a concentration, which results in a non-uniform electric field distribution inside the PDLCs, limits the free steering of liquid crystal molecules under the electric field and reduces the dipole moment response, which leads to a slight decrease in the dielectric constant. The effective contact area of the agglomerated CNCs will be reduced. At this time, the dielectric loss is lower than that of samples with low concentrations of doping but still higher than that of the undoped sample.

**Figure 6:**
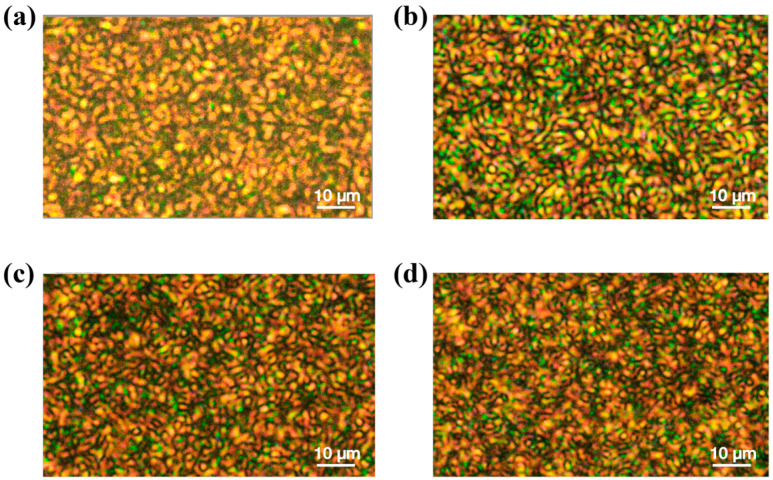
Effect of KH560-modified CNCs concentration on liquid crystal droplet morphology studied using POM: (**a**) 0 wt%, (**b**) 0.02 wt%, (**c**) 0.2 wt%, (**d**) 0.5 wt%.

Figure 6 shows samples with different concentrations of CNC observed using a polarized light microscope. By comparing Figure 6a–d, it can be seen that the liquid crystal droplets are smaller in Figure 6d.

**Figure 7:**
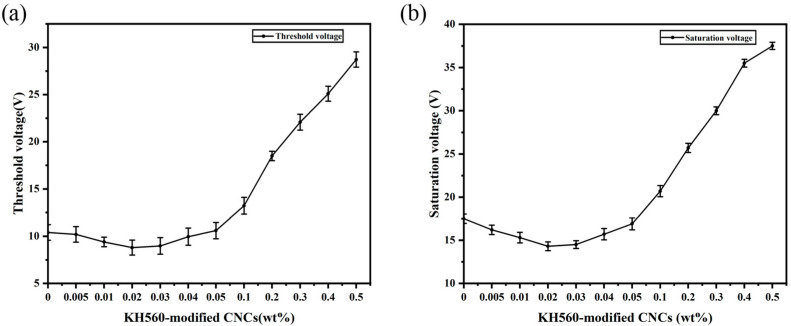
Threshold voltage and saturation voltage curves of PDLC films doped with CNC: (**a**) threshold voltage; (**b**) saturation voltage.

The detailed data for the threshold voltage and saturation voltage of all PDLC samples are shown in Figure 7a,b. Both the threshold voltage and the saturation voltage show a nonlinear relationship with the doping concentration: as the concentration increases, both voltages first decrease and then increase, with the lowest value of the threshold voltage reaching 8.8±0.8 V and the lowest value of the saturation voltage being 14.4±0.5 V, corresponding to a CNC doping concentration of 0.02 wt%.

This can be attributed to the fact that without CNC doping, the liquid crystal microdroplets in PDLC may be randomly distributed. The introduction of a small amount of CNC promotes the ordered arrangement of liquid crystal molecules in the electric field. Since CNC is a one-dimensional nanorod-like structure, the helical arrangement of its molecular chains endows it with intrinsic chiral characteristics [48]. The fibrous structure of CNC can act as a guiding template for the nucleation of liquid crystal molecules. By guiding liquid crystal molecules to aggregate at these nucleation points with chirality, liquid crystal microdroplets interact with the chiral center of CNC to form an ordered liquid crystal phase. This ordered arrangement enhances the alignment efficiency of liquid crystal molecules under the electric field, reducing the required electric field strength for their rearrangement. Although the size of the liquid crystal microdroplets does not change significantly, the voltage is usually proportional to the dielectric constant. In the PDLC system, samples with low concentrations of CNC exhibit higher dielectric constants and stronger conductivity, which also leads to lower voltages for these samples.

However, the situation is different when the doping concentration of CNC is higher, and a higher concentration of CNC tends to aggregate and form larger particles or network structures. The formation of aggregates not only causes the arrangement of liquid crystal molecules in the polymer matrix to become irregular but also interferes with the normal arrangement of liquid crystal molecules, resulting in the formation of aggregates to modify the polymer matrix, which, in turn, requires a greater electric field strength for the reorientation process of the liquid crystal molecules [49]. This requires the application of a more potent electric field force to overcome the anchoring effect of the polymer on the liquid crystals, inducing the reorientation of the liquid crystal microdroplets in the direction of the applied electric field. *V* is given by the following equation:

(2) $$\;\;\;V=\frac{d}{R}\sqrt{\frac{K\left(l^{2}-1\right)}{\varepsilon_{0}\Delta \varepsilon }}$$

where *d*, *K*, *R*, *l* and *Δε* are the film thickness, effective elastic constant, liquid crystal microdroplets radius, aspect ratio of liquid crystal microdroplets and dielectric anisotropy.

The voltage is inversely proportional to the liquid crystal droplet size. At higher doping concentrations, the liquid crystal droplet size decreases and the number of droplets increases (Figure 6c,d), and then the droplets’ refinement significantly affects the voltage [50,51,52]. This ultimately leads to higher threshold and saturation voltages for PDLCs with higher doping concentrations.

The equation extended by Owens and Wendt is as follows [53]:

(3) $$\frac{1+\cos\theta }{2}\times \frac{\gamma_{l}}{\sqrt{\gamma_{l}^{d}}}=\sqrt{\gamma_{s}^{p}}\times \sqrt{\frac{\gamma_{l}^{p}}{\gamma_{l}^{d}}}+\sqrt{\gamma_{s}^{d}}\;$$

where $\gamma_{l}$ is the surface energy of liquid, $\gamma_{l}^{p}\;$ is polar component of liquid surface energy, $\gamma_{l}^{d}\;$ is dispersion component of liquid surface energy, $\gamma_{s}^{p}$ is polar component of solid surface energy and $\gamma_{s}^{d}$ is dispersion component of liquid surface energy.

The Owens and Wendt equation is in this form:

(4) y=a·x+b

Substituting the parameters of Table 1 into the Owens and Wendt equation, the slope and intercept obtained through fitting are *a* = 2.643, *b* = 5.843. Hence, the total surface energy of the polymer is calculated as follows:

(5) $$\gamma_{s}=\gamma_{s}^{d}+\gamma_{s}^{p}=a^{2}+b^{2}=41.13\;mJ/m^{2}$$

The surface energy of E7 LC was calculated by adding E7 liquid crystal to the polymer, and the measured contact angle (θ) was 30 degrees.

Then, use Neumann’s equation [56]:

(6) $$1+\cos\theta =2\left(\frac{\gamma_{s}}{\gamma_{l}}\right)e^{-0.0001247({\gamma_{l}-\gamma_{s})}^{2}}$$

So, the surface energy $\gamma_{l}$ of E7 liquid crystal is 44.04 $mJ/m^{2}$.

Then, use the Young–Dupre equation to calculate the E7 on polymers doped with different concentrations of KH560-modified CNCs’ interface energy [57]:

(7) $$\;\;\;w_{a}=\gamma_{l}\left(1+\cos\theta \right)$$

**Figure 8:**
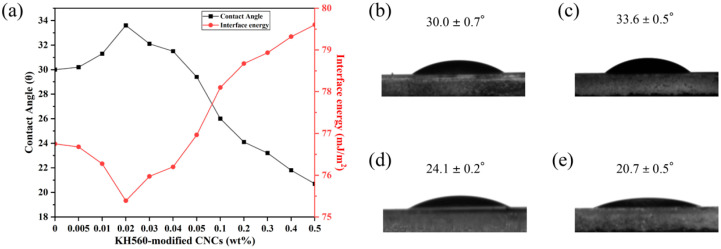
(**a**) The variation in contact angle and interfacial energy of E7 on polymer surface with the addition of KH560-modified CNCs; contact angle measurements of E7 on polymers doped with various concentrations of KH560-modified CNCs: (**b**) 0 wt%, (**c**) 0.02 wt%, (**d**) 0.2 wt%, (**e**) 0.5 wt%.

The changes in contact angle and interfacial energy of E7 on the polymer surface after the addition of KH560-modified CNCs are shown in Figure 8, where the contact angle increases from the initial 30 degrees to 33.6 degrees and then decreases to 20.7 degrees. With the increase in doping concentration, the interfacial energy initially decreases and then increases. The anchoring effect of the polymer on the liquid crystal is also shown to decrease and then increase.

As shown in Figure 8, the smaller concentration of CNC added makes the interfacial energy of the polymer–liquid crystal decrease. It has smaller anchoring energy. Samples with lower interfacial energy have weaker interactions between the polymer and liquid crystals. This interfacial modulation helps stabilize the liquid crystal microdroplets and reduce the microdroplet deformation or aggregation due to interfacial interactions. Meanwhile, the amphiphilic KH560-modified CNCs enhance the dispersion in the lipophilic matrix and act as a lubricant at the boundary.

**Figure 9:**
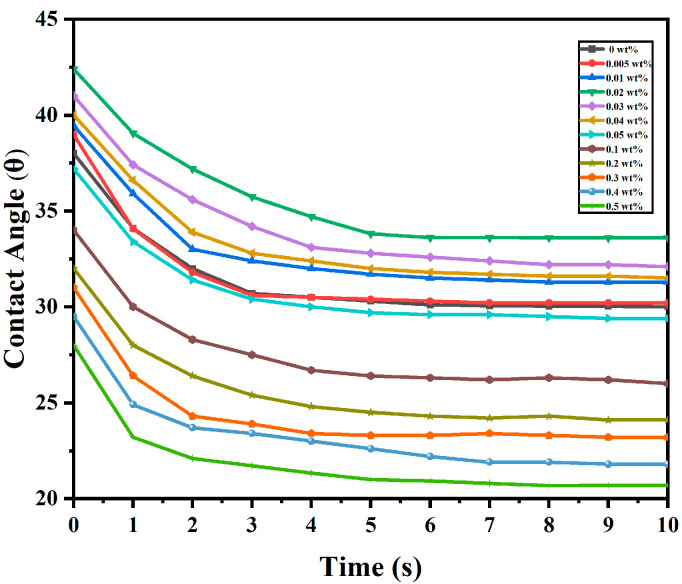
The kinetic evolution of the contact angle for E7 liquid crystal with various concentrations of KH560-modified CNCs.

Figure 9 illustrates the dynamic evolution of the contact angle between the E7 liquid crystal with various concentrations of KH560-modified CNCs at the polymer, with measurements taken at 0–10 s.

Figure 9 shows that the E7 liquid crystal with 0.02 wt% concentrations of KH560-modified CNCs exhibits slower temporal evolution of the contact angle. It can be observed that at lower KH560-CNC concentrations, the contact angle decreases at a slower rate, requiring a longer time to reach equilibrium [58]. From Figure 8, the E7 with a low concentration of KH560-CNCs has a lower interface energy, which suppresses the spreading dynamics of the liquid crystal molecules. In contrast, higher KH560-modified CNC concentrations increase the interface energy, resulting in a faster decrease in the contact angle and a shorter time required to reach equilibrium from the initial contact angle.

However, with the increasing doping concentration, the high crystallinity of CNC implies a highly ordered arrangement of cellulose molecular chains on the nanoscale, as well as the surface being rich in hydroxyl groups. When aggregation occurs, hydrogen bonding binds the CNC tightly, which, in turn, reduces its interaction with the polymer matrix. Aggregated CNC reduces the effective contact area and also causes interfacial defects, leading to an increase in interfacial energy, which enhances the anchoring effect of the polymer on the liquid crystal. It also enhances the interaction between the polymer and the liquid crystal. At the same time, CNC aggregates become more stable due to weak intermolecular forces, which increase surface friction, similar to the behavior of other surfactants [59].

**Figure 10:**
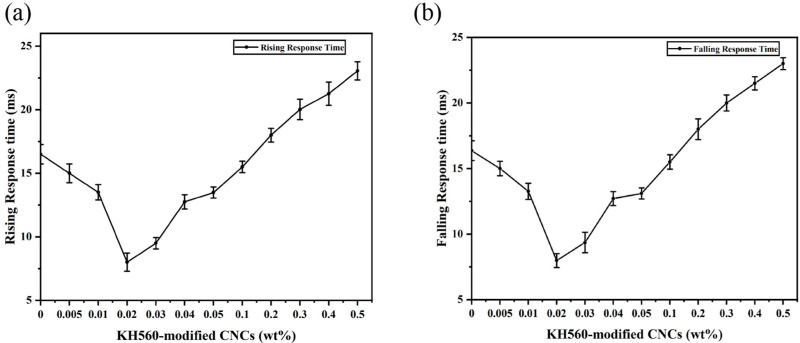
Rise response time and fall response time of PDLC films doped with CNC: (**a**) rise response time; (**b**) fall response time.

Figure 10 shows the rise time and fall time of PDLC samples with varying doping concentrations. As can be seen from the figure, with the increase in CNC doping concentration, the rise time and fall time of the test samples showed a trend of decreasing and then increasing, with the lowest fall time of 8±0.7 ms and a rise time of 16.5±0.5 ms. The changes in the rise time and fall time reflect the effect of KH560-modified CNC doping on the orientation of liquid crystals and the interaction of liquid crystals with the polymer matrix.

The rise time is mainly dependent on the driving of the liquid crystal molecules by the applied voltage. It is less related to the interaction between the liquid crystals and the polymer. When the concentration of doped CNC is lower, the fibrous structure of CNC guides the liquid crystal molecules to form an ordered structure within the polymer matrix. Additionally, CNC acts as a lubricant at the boundary. These two effects accelerate the response of the liquid crystal molecules to the electric field, resulting in a decrease in the rise time. As the doping concentration increases, the increase in interfacial energy results in a corresponding increase in rise time [60].

The fall time is a key indicator of the interaction between the liquid crystal and the polymer matrix. When the voltage is removed, the liquid crystal molecules must overcome the friction between the liquid crystal and the polymer, known as the interfacial resistance, to return to their original state. This resistance results from the interaction between the two and prevents the liquid crystal molecules from rotating. When doped with CNC at a low concentration, CNC plays the role of a “lubricant” at the boundary, effectively reducing the “frictional resistance” in the recovery process of the liquid crystal molecules, thereby accelerating the response speed of the liquid crystal molecules and shortening the fall time. However, as the doping concentration increases, CNC aggregation occurs, leading to increased interfacial energy of the polymer–liquid crystal and enhancement of polymer–liquid crystal bonding strength, so that the recovery of liquid crystal molecules is blocked. This aggregation causes the liquid crystal molecules to take longer to recover to the random state, which ultimately leads to an increase in the fall time.

**Figure 11:**
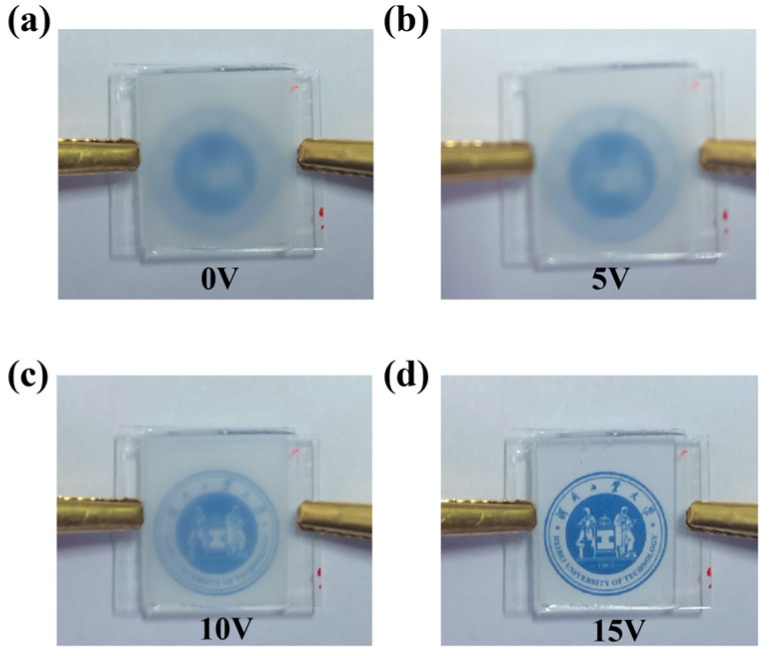
Photographs of the PDLC film with 0.02 wt% CNCs film at different voltages: (**a**) 0 V; (**b**) 5 V; (**c**) 10 V; (**d**) 15 V.

Figure 11 presents images of the PDLC film with 0.02 wt% CNC under different voltages. Without voltage the film is opaque. At 5 V, the background becomes slightly visible; at 10 V, the transmittance is further enhanced, and at 15 V, the background is clearly visible.

Then, as shown in Table 2, the threshold voltage, saturation voltage, current ratio, response speed and their respective rates of change relative to the undoped system were quantitatively compared with other doped nanoparticle systems [61,62,63,64,65,66,67,68,69,70,71]. The optimal performance metrics of this study are as follows: *V*th 8.8 ± 0.8 V, *V*_sat_ 14.4 ± 0.5 V, CR 152 and response time 8±0.7 ms. Compared to other doped systems, this study offers the advantages of low threshold voltage (<10 V) and fast response (<10 ms).

## 3. Experiments

### 3.1. Materials

In this experiment, E7 liquid crystal (*n*_o_ = 1.520, *n*_e_ = 1.747, Δ*n* = 0.227) purchased from Jiangsu Hecheng Display Technology Co. (Nanjing, China) was used. The acrylate monomer used in the experiment was isononyl acrylate (INAA, *n* = 1.444), which was provided by Changxing Chemical Materials Co. (Chengdu, China). The photoinitiator was PI 184 manufactured by Shanghai Yinchang New Materials Co. (Shanghai, China). For the substrate, the other materials included prepolymer CN131, isobornyl acrylate (IBOA, *n* = 1.474) and isobornyl methacrylate (IBOMA, *n* = 1.474). CN131 was purchased from Shadoma Ltd. (Guangzhou, China), and IBOA and IBOMA were provided by Changxing Chemical Materials Co. (Chengdu, China). The dopant material chosen for the experiment was silane-modified cellulose nanocrystals (KH560-modified CNCs) with a length of 146 nm and a diameter of 5 nm, purchased from Tianjin Wood Wizard Biotechnology Co. (Tianjin, China).

### 3.2. Functionalization of KH560-Modified CNCs

**Figure 12:**
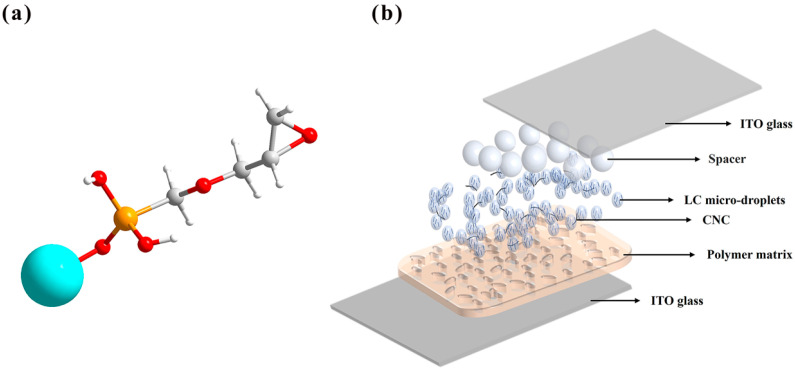
(**a**) Ball-and-stick model of modified functional groups. (**b**) Detailed structural schematic diagram of CNC-doped PDLC.

In the experiments, KH560-modified CNCs were used to prepare CNC-modified solutions. First, 200 mL of anhydrous acetone were measured as the solvent, and 10 g of KH560-modified CNCs were weighed. Using a magnetic stirrer, the particles were slowly added to the acetone and continuously stirred for 2 h to ensure uniform dispersion in the solvent. To further improve the dispersion, the solution was treated with an ultrasonic oscillator for 4 h to break the aggregated state of the particles and ensure the stability of the suspension. A final 5% CNC-modified solution was obtained. Figure 12a shows the ball-and-stick model of the modified CNC.

CNC has good hydrophilicity, which can be attributed to the presence of a large number of polar functional groups, such as hydroxyl (−OH), in its molecular structure. These polar functional groups are able to form hydrogen bonds with water molecules, thus facilitating the interaction between CNC and water and enabling CNC to absorb water. By modifying the surface of nanocellulose with the silane coupling agent KH560, the silane group (-Si(OCH_3_)_3_) in the KH560 molecule undergoes a hydrolytic condensation reaction with some of the hydroxyl groups on the surface of nanocellulose, forming stable silicon–oxygen bonds (Si-O-C) and releasing methanol and water molecules. After KH560 modification, hydrophobic functional groups, such as silanol groups (-Si(OH)_2_) and epoxy groups, were introduced on the surface of CNC. The CNCs were further modified with KH560 with the aim of introducing both hydrophilic and lipophilic properties on the CNC surface, which promotes that CNCs can be homogeneously dispersed in PDLC systems due to the difference in chemical properties between polymer–liquid crystals and KH560-modified CNCs in PDLC systems.

Figure 12a refers to the ball-and-stick model of modified functional groups, where the blue ball refers to CNC, the yellow ball refers to silicon atoms, the red one refers to oxygen atoms, the smaller white ball refers to hydrogen atoms and the larger white ball refers to carbon atoms. The schematic diagram of the PDLC thin film after the addition of KH560-modified CNCs is shown in Figure 12b. The ITO glass substrate is sandwiched with polymer, liquid crystal microdroplets, CNCs and spacers.

### 3.3. Preparation of the PDLC Films

In this experiment, PDLC films were prepared by the polymerization-initiated phase separation method. The compositions of the samples are shown in Table 3, including E7 liquid crystal and CN131, INAA, IBOA, IBOMA and photoinitiator 184, in precise mass percentages.

Initially, the polymer was proportionally mixed with the liquid crystals in a small brown bottle, and the prepared CNC-modified solution was doped on this basis. Subsequently, ultrasonic dispersion was carried out for 4 h to ensure that the CNCs were sufficiently dispersed in the polymer liquid crystals, so that the energy from ultrasonic oscillation could also evaporate the acetone to form a homogeneous initial mixture.

**Figure 13:**
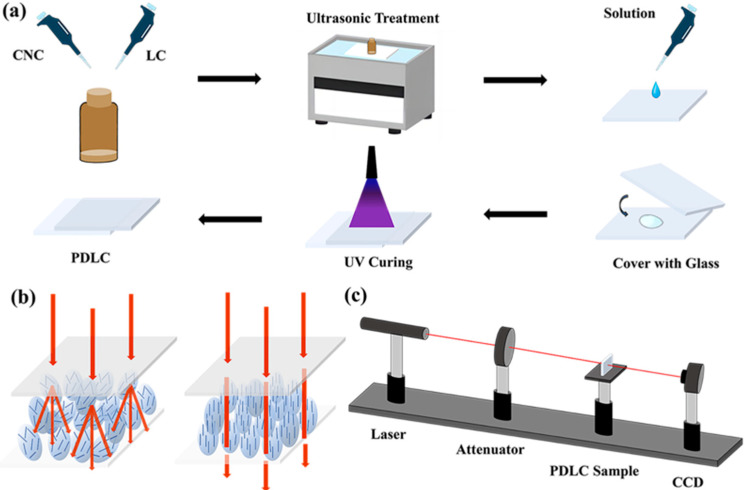
(**a**) Preparation process of PDLC film. (**b**) Schematic illustration of the working mechanism of the PDLC. (**c**) Illustration of the experimental instruments.

Thereafter, an ITO electrode glass (2 cm × 2 cm) was cleansed with anhydrous ethanol and subsequently sprinkled with 10 µm sized spacers. Then, 4 µL of the initial mixture were added dropwise with a pipette gun, and the other glass was slowly covered. Subsequently, it was placed under UV-A light for 90 s (light intensity 12 mW/cm^2^) with a total exposure time of 3 min to complete the preparation of PDLC films. The preparation process is shown in Figure 13a.

Eleven PDLC film samples with different doping ratios of CNC-modified solutions were prepared, and one PDLC sample with undoped CNC-modified solution was also prepared to compare the results. Twelve PDLC film samples were prepared with varying KH560-modified CNCs doping concentrations, with the full experimental conditions detailed in Table 4.

In the absence of an electric field, the liquid crystal microdroplets are dispersed within the polymer matrix, resulting in a scattering state for the film. Conversely, when an electric field is applied, the liquid crystal microdroplets become ordered along the direction of the electric field, thereby transitioning the film to a light-transmitting state, as illustrated in Figure 13b.

### 3.4. Characterization

Characterization of electro-optic (EO): The electro-optical parameters of liquid crystals were measured using an optical system, as shown in Figure 13c. A He-Ne laser (DH-HN250P) with a wavelength of 632.8 nm was used as the light source. The sample was located 32 cm behind the laser. Subsequent to the laser’s traversal of the sample, a 1 cm × 1 cm photodetector (Dsi300) was positioned at a distance of 30 cm behind the sample to receive the light transmitted through the sample. The transmittance of the samples is normalized by the transmittance of air as 100%, and the transmittance of the samples is obtained by dividing the transmittance values of different samples by the transmittance value of air to eliminate the influence of other light in the environment. The sample was driven using a 1 kHz square wave alternating current with a voltage step of 1 V. The transmittance–voltage curve was collected and processed by a computer, and the response time of liquid crystals to the voltage change was measured by an oscilloscope (DS1104Z-S). Each sample was measured at least 5 times, and the average value was taken.

Characterization of dielectric: The dielectric constant and loss tangent of the samples were measured using a precision LCR meter (AgilentE4980A) in the frequency range of 100–10,000 Hz at 1 V (Keysight, Santa Rosa, CA, USA).

Characterization of polarizing optical microscope (POM): The polarizing optical microscope (POM) image of the sample at different voltages was observed through an orthogonal polarizer.

Characterization of contact angle: The contact angles were measured using a surface tension analyzer (DSA-100), which is equipped with a high-speed CCD image sensor (60 fps). This instrument captures the instantaneous patterns of the contact angle between liquid and solid surfaces through a high-resolution optical imaging system and precise liquid dispensing setup. First, mixtures of E7 liquid crystal with various concentrations of KH560-modified CNCs were prepared, followed by dropping 5 uL of the polymer solution onto a slide and covering it with PET film, which was removed after 90 s of curing under UV light, to obtain a flat film containing the polymer. Then, the contact angles of E7 liquid crystal with various concentrations of KH560-modified CNCs, water, ethylene glycol and diiodomethane droplets with the cured polymer surface at 10 s were measured.

Characterization of viewing angle: The viewing angle properties of the PDLC films were characterized by measuring the variation of the transmittance corresponding to the threshold voltage with the angle between the laser beam and the sample using a rotary stage to control the angle from 0 to 60 degrees in 10-degree increments in order to evaluate the optical properties at different viewing angles.

### 3.5. Definition of Electro-Optical Performance Parameters

In this experiment, the electro-optical performance parameters of PDLC films were characterized by plotting the transmittance versus voltage curve of PDLC samples. From this curve, parameters including threshold voltage (*V*th), saturation voltage (*V*_sat_), maximum transmittance (*T*_max_) and minimum transmittance (*T*_min_) could be directly read. *V*th was defined as the minimum voltage required to produce a significant change in the transmittance, corresponding to a voltage when the transmittance was *T*_10%_, and *V*_sat_ was the voltage required to achieve the maximum transmittance, corresponding to a voltage when the transmittance was *T*_90%_. *T*_10%_ = (*T*_max_ − *T*_min_) × 10% + *T*_min_ and *T*_90%_ = (*T*_max_ − *T*_min_) × 90% + *T*_min_. The contrast ratio, an important indicator of the electro-optical performance, was defined as the ratio of the maximum transmittance to the minimum transmittance. The rise time (*t*_on_) was defined as the time required for the transmittance to rise from *T*_10%_ to *T*_90%_ after applying a voltage. Similarly, the fall time (*t*_off_) was the time required for the transmittance to fall from *T*_90%_ to *T*_10%_ after the voltage was switched off. These parameters were used to describe the response behavior and electro-optical properties of PDLC samples at different voltages.

## 4. Conclusions

In this study, we investigated the effect of KH560-modified CNC doping on the electro-optical properties of PDLC and proposed the mechanism of CNC in PDLC. It was found that low concentrations (0.005–0.05 wt%) of CNC doping significantly improved the electro-optical properties of PDLC. At 0.02 wt% concentration, the threshold and saturation voltages were reduced, the response speed was increased by about 30% and the viewing angle performance was broadened, which was mainly due to the interaction of the fiber structure of CNC with liquid crystals to enhance the alignment effect and the homogeneous dispersion at a low concentration to reduce randomness and light scattering. However, as the concentration of CNC increases, the aggregation phenomenon interferes with the alignment of liquid crystals, resulting in higher threshold and saturation voltages, longer response time and lower light transmission. A limitation of this study is its narrow optimal concentration window (0.005–0.05 wt%), indicating that the CNC doping process has not yet been validated for the large-scale production of PDLCs. Moreover, when the concentration exceeds 0.05 wt%, the relationship between the number and type of CNC aggregates and liquid crystal defects remains unclear. This necessitates further research into CNC surface modification or composite doping strategies to stabilize the interaction between CNC and liquid crystal polymer systems. Additionally, these findings should be extended to other liquid crystal systems, and predictive models for the morphology and electro-optical properties of CNC should be established, all of which will drive the development of smart dimming devices.

## Figures and Tables

**Table 1:** Surface tension parameters for various liquids and the contact angle on the polymer [54,55].

**Liquid**	$\gamma_{l\;}$ **(** $mJ/m^{2}$ **)**	$\gamma_{l}^{d\;}$ **(** $mJ/m^{2}$ **)**	$\gamma_{l}^{p\;}$ **(** $mJ/m^{2}$ **)**	Contact Angle on Polymer (θ)
Water	72.8	21.8	51.0	74.1
Ethylene glycol	48.2	29.0	19.2	40.2
Diiodomethane	50.8	48.5	2.3	39.2

**Table 2:** The comparison of the threshold voltage, saturation voltage, current ratio and response speed in this work with other work in the literature.

**Materials**	$V_{th\;}$ **(V)**	$\Delta V_{th\;}$ **(V)**	$V_{sat}$ **(V)**	$\Delta V_{sat}$ **(V)**	*CR*	ΔCR	$t_{off}$ **(ms)**	$\Delta t_{off}$ **(ms)**	**Ref.**
Span-80	5	decrease 33%	12	decrease 29%	-	-	-	-	[61]
ZrO_2_	20	decrease 20%	39	decrease 18%	73	increase 40%	280	increase 115%	[62]
DBS	16	decrease 35%	21	decrease 46%	205	increase 25%	488.3	increase 163%	[63]
CNC	8.8	decrease 15%	14.4	decrease 19%	152	increase 7%	8	decrease 52%	This work
ZnA	10	decrease 50%	20	decrease 33%	7	increase 27%	8.1	decrease 8%	[64]
KH570-SiO_2_	5	decrease 81%	21	decrease 63%	159	increase 33%	180	increase 92%	[65]
MNPs	3.53	decrease 58%	14.6	decrease 19%	14.3	increase 48%	-	-	[66]
silica NPs	3.7	decrease 62%	8	decrease 73%	-	-	-	-	[67]
TiO_2_/ MPTS	16	increase 77%	30	increase 25%	97	increase 177%	10	decrease 82%	[68]
WO_3_ NPs	13.6	decrease 33%	20	decrease 32%	194	increase 84%	-	-	[69]
γ-Fe_2_O_3_ MNPs	9.5	decrease 33%	22	decrease 19%	-	-	-	-	[70]
AuNPs	6.5	decrease 13%	34	decrease 5.5%	3	decrease 2%	-	-	[71]

**Table 3:** The compositions of liquid crystal and polymer.

**Polymer Composition (wt%)**		**E7 (wt%)**
INAA	6.8	60
IBOA	9.6	
IBOMA	9.6	
CN131	12.8	
PI 184	1.2	

**Table 4:** The composition and preparation conditions of PDLC films with KH560-modified CNCs.

**Polymer: LC** **(wt%)**	**CNC (wt%)**	**UV-A Curing**		**Ultrasonic Dispersion**		**Magnetic Stirring** **Time (h)**	Temperature (°C)
		**Intensity (mW/cm^2^)**	**Time (min)**	**Time (h)**	Temperature (°C)		
40:60	0	12	1.5	4	60	2	25
	0.005						
	0.01						
	0.02						
	0.03						
	0.04						
	0.05						
	0.1						
	0.2						
	0.3						
	0.4						
	0.5						

## Data Availability

Data are contained within the article.
